# Information Theoretic Approaches for Motor-Imagery BCI Systems: Review and Experimental Comparison

**DOI:** 10.3390/e20010007

**Published:** 2018-01-02

**Authors:** Rubén Martín-Clemente, Javier Olias, Deepa Beeta Thiyam, Andrzej Cichocki, Sergio Cruces

**Affiliations:** 1 Departamento de Teoría de la Señal y Comunicaciones, Universidad de Sevilla, Camino de los Descubrimientos s/n, 41092 Seville, Spain; 2 Department of Sensor and Biomedical Technology, School of Electronics Engineering, VIT University, Vellore, Tamil Nadu 632014, India; 3 Skolkovo Institute of Science and Technology (Skoltech), Moscow 143026, Russia; A.Cichocki@skoltech.ru or; 4 Laboratory for Advanced Brain Signal Processing, Brain Science Institute, RIKEN, 2-1 Hirosawa, Wako, Saitama 351-0198, Japan; 5 Systems Research Institute, Polish Academy of Sciences, 01-447 Warsaw, Poland

**Keywords:** common spatial patterns, generalized divergences, brain computer interfaces

## Abstract

Brain computer interfaces (BCIs) have been attracting a great interest in recent years. The common spatial patterns (CSP) technique is a well-established approach to the spatial filtering of the electroencephalogram (EEG) data in BCI applications. Even though CSP was originally proposed from a heuristic viewpoint, it can be also built on very strong foundations using information theory. This paper reviews the relationship between CSP and several information-theoretic approaches, including the Kullback–Leibler divergence, the Beta divergence and the Alpha-Beta log-det (AB-LD)divergence. We also revise other approaches based on the idea of selecting those features that are maximally informative about the class labels. The performance of all the methods will be also compared via experiments.

## 1. Introduction

The electroencephalogram (EEG) is a record over time of the differences of potential that exist between different locations on the surface of the head [1,2]. It originates from the summation of the synchronous electrical activity of millions of neurons distributed within the cortex. In recent years, there has been a growing interest in using the EEG as a new communication channel between humans and computers. Brain-computer interfaces (BCIs) are computer-based systems that enable us to control a device with the mind, without any muscular intervention [3,4,5,6]. This technology, though not yet mature, has a number of therapeutic applications, such as the control of wheelchairs by persons with severe disabilities, but also finds use in fields as diverse as gaming, art or access control.

**Figure 1:**
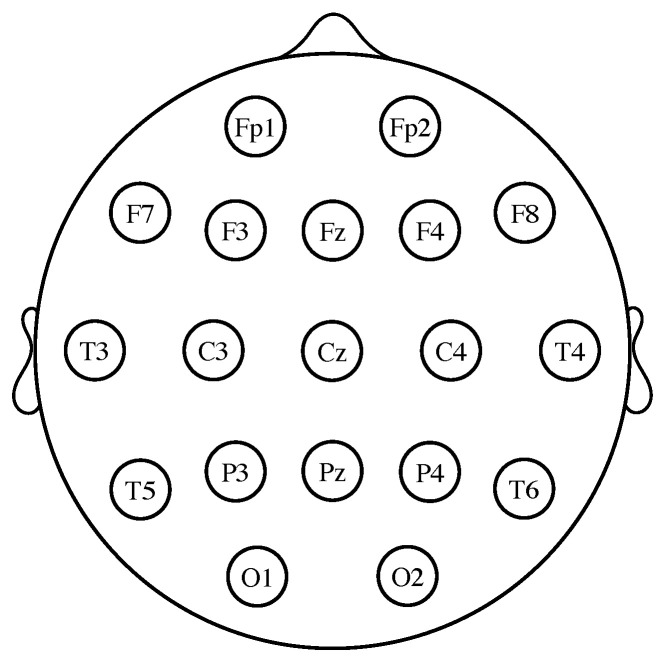
Electrode locations of the international 10–20 system for EEG recording. The letters “F”, “T”, “C”, “P” and “O” stand for frontal, temporal, central, parietal and occipital lobes, respectively. Even numbers correspond to electrodes placed on the right hemisphere, whereas odd numbers refer to those on the left hemisphere. The “z” refers to electrodes placed in the midline.

There are several possible approaches for designing a BCI [1,4,7]. Among them, motor imagery (MI)-based BCI systems seem to be the most promising option [6,8,9,10]. In MI-based BCI systems, the subject is asked to imagine the movement of different parts of his or her body, such as the hands or the feet. The imagined actions are then translated into different device commands (e.g., when the subject imagines the motion of the left hand, the wheelchair is instructed to turn to the left). What makes this possible is that the spatial distribution of the EEG differs between different imagined movements. More precisely, since each brain hemisphere mainly controls the opposite side of the body, the imagination of right and left limb movements produces a change of power over the contralateral left and right brain motor areas. These fluctuations, which are due to a pair of phenomena known as event-related desynchronization (ERD) or power decrease and event-related synchronization (ERS) or power increase [11,12], can be detected and converted into numerical features. By repeating the imagined actions several times, a classifier can be trained to determine which kind of motion the subject is imagining (see [13] for a review). In practice, three classes of MI are used in BCIs, namely the movements of the hands, the feet and the tongue. Left hand movement imagery is more prominent in the vicinity of the electrode C4 (see Figure 1), while right hand imagined actions are detected around electrode C3 [14]. The imagery of feet movements appears in the electrode Cz and its surrounding area; nevertheless, it is not usually possible to distinguish between left foot or right foot motor imagery because the corresponding activation areas are too close in the cortex [11,14]. Finally, imagery of tongue movements can be detected on the primary motor cortex and the premotor cortex [15]. One of the inherent difficulties of designing a BCI is that the EEG features are highly non-stationary and vary over sessions. To cope with this problem, the background state of the subject (i.e., his or her motivation, fatigue, etcetera) and the context of the experiment can be both modeled as latent variables, whose parameters can be estimated using the expectation-maximization (EM) algorithm [16,17]. Overall, current BCI approaches achieve success rates of over 90%, although much depends on the person from whom the EEG data are recorded [14].

The common spatial patterns (CSP) method [4,18,19,20,21,22] is a method of dimensionality reduction that is widely used in BCI systems as a preprocessing step. Basically, assuming two classes of MI-EEG signals (e.g., left hand and right hand MI tasks), CSP projects the EEG signals onto a low-dimensional subspace, which captures the variability of one of the classes while, at the same time, trying to minimize the variance in the other class. The goal is to enhance the ability of the BCI to discriminate between the different MI tasks, and it has been shown that CSP is able to reduce the dimension of the data significantly without decreasing the classification rate. It is noteworthy that CSP admits an interesting probabilistic interpretation. Under the assumption of Gaussian distributed data, CSP is equivalent to maximizing the symmetric Kullback–Leibler (KL) divergence between the probability distributions of the two classes after the projection onto the low dimensional space [23,24]. As a generalization of this idea in the context of BCI, it is interesting to investigate the dimensionality reduction ability of other different divergence-based criteria, which is drawing a lot of interest among the computational neuroscience community.

The present manuscript is a review of the state of the art of information theoretic approaches for motor imagery BCI systems. The article is written as a guideline for researchers and developers both in the fields of information theory and BCI, and the goal is to simplify and organize the ideas. We will present a number of approaches based on Kullback–Leibler divergence, Beta divergence (which is a generalization of Kullback–Leibler’s) and Alpha-Beta log-det (AB-LD) divergence (which include as special cases Stein’s loss, the *S*-divergence or the Riemannian metric), as well as their relation to CSP. We will also review a technique based on the idea of selecting those features that are maximally informative about the class labels. Complementarily, for the purpose of comparison, several non-information theoretic variants of CSP and their different regularization schemes are revised in the paper. The performance of all approaches will be evaluated and compared through simulations using both real and synthetic datasets.

The paper is organized as follows: The CSP algorithm is introduced in Section 2. Section 3 introduces the main characteristics of the Kullback–Leibler divergence, the Beta divergence and the Alpha-Beta log-det divergence, respectively, as well as their application to the problem of designing MI-BCI systems and the algorithms used to optimize them. Section 4 reviews an information-theoretic feature extraction framework. Section 5 presents, as has been said before, several extensions of CSP not based on information-theoretic principles. Finally, Section 6 presents the results of some experiments in which the performances of the above criteria are tested, in terms of their accuracy, computational burden and robustness against errors.

### EEG Measurement and Preprocessing

For measuring the EEG, several different standardized electrode placement configurations exist. The most common among them is the International 10–20 system, which uses a set of electrodes placed at locations defined relative to certain anatomical landmarks (see Figure 1). The ground reference electrode is usually positioned at the ears or at the mastoid. To obtain a reference-free system, it is common practice to calculate the average of all the electrode potentials and subtract it from the measurements [1,2].

The EEG is usually contaminated by several types of noise and artifacts. Eye blinks, for example, elicit a large potential difference between the cornea and the retina that can be several orders of magnitude greater than the EEG. In the rest of the paper, it is assumed that the signals have already been pre-processed to remove noise and interferences. To this end, several techniques [25], such as autoregressive modeling [26], the more complex independent component analysis (ICA) [27], or the signal space projection (SSP) method [28], have shown good or excellent results (see also [29] and the references therein). Signal preprocessing includes also the division of the EEG into several frequency bands that are separately analyzed [30,31]. The “mu” band (8–15 Hz) and the “beta” band (16–31 Hz) are particularly useful in BCIs, as they originate from the sensorimotor cortex, i.e., the area that controls voluntary movements [2].

## 2. The Common Spatial Pattern Criterion

In this section, we present the common spatial patterns (CSP) method [4,18,19,20,21,22,32,33]. Consider a two-class classification problem, where the EEG signals belong to exactly one of two classes or conditions (e.g., left-/right-hand movement imagination).

To fix notation, let $X_{i,k}\in R^{D\times T}$ be the matrix that contains the EEG data of class i∈{1,2} in the *k*-th trial or experiment, where *D* is the number of channels and *T* the number of samples in a trial. The corresponding sample covariance estimator is defined by:

(1) $$\begin{array}{c} \Sigma_{i,k}=\frac{1}{T-1}X_{i,k}\;X_{i,k}^{⊤}, \end{array}$$

where ${\left(\cdot \right)}^{⊤}$ denotes “transpose”. Here, the EEG signals are assumed to have zero-mean, which is fulfilled as they are band-pass filtered (see the previous section). If *L* trials per class are performed, the spatial covariance matrix for class *i* is usually calculated by averaging the trial covariance matrices as:

(2) $$\begin{array}{c} \Sigma_{i}=\frac{1}{L}\sum_{k=1}^{L}\Sigma_{i,k} \end{array}$$

In practice, these covariance matrices are often normalized in power with the help of the following transformation:

(3) $$\begin{array}{c} \Sigma_{i}\leftarrow \Sigma_{i}/\mathrm{tr}\left(\Sigma_{i}\right), \end{array}$$

where tr(·) denotes the trace operator.

After the BCI training phase, in which matrices $\Sigma_{1}$ and $\Sigma_{2}$ are estimated using training data, suppose that a new, not previously observed, data matrix $X\in R^{D\times T}$ of imagined action is captured. The problem that arises is to develop a rule to allocate these new data to one class or the other. A useful approach is to define a weight vector $w\in R^{D}$ (also known as a ‘spatial filter’) and allocate X to one class if the variance of $w^{⊤}X$ exceeds a certain predefined threshold and to the other if not; this relates to the fact that event-related desynchronizations and event-related synchronizations, i.e., the phenomena underlying the MI responses, are associated with power decreases/increases of the ongoing EEG activity [12].

Of course, not just any spatial filter is of value. To enhance the discrimination of the MI tasks, CSP proposes using spatial filters that maximize the variance of the band-pass filtered EEG signals in one class while, simultaneously, minimizing it for the other class. Mathematically, CSP aims at maximizing an objective function based on the the following Rayleigh quotient:

(4) $$\begin{array}{cc} J(w) & =\frac{w^{⊤}\Sigma_{1}w}{w^{⊤}\Sigma_{2}w}=\frac{\sigma_{1}^{2}}{\sigma_{2}^{2}}, \end{array}$$

where $\sigma_{i}^{2}$ is the variance of the *i*-th projected class and $\Sigma_{i}$ is the covariance matrix of the *i*-th class.

It is a straightforward derivation to obtain that the spatial filters that hierarchically maximize (4) can be computed by solving the generalized eigenvalue problem:

(5) $$\Sigma_{1}w=\lambda \Sigma_{2}w.$$

Each eigenvector $w_{i}$ gives a different solution. Observe that:

$$w_{i}^{⊤}\Sigma_{1}w_{i}=\lambda_{i}w_{i}^{⊤}\Sigma_{2}w_{i}\to \lambda_{i}=\frac{w_{i}^{⊤}\Sigma_{1}w_{i}}{w_{i}^{⊤}\Sigma_{2}w_{i}}=J\left(w_{i}\right),$$

where $\lambda_{i}$ is the generalized eigenvalue corresponding to $w_{i}$. Therefore, the larger (or smaller) the eigenvalue, the larger the ratio between the variances of the two classes and the better the discrimination accuracy of the filter.

The latter readily suggests selecting the spatial filters among the principal and the minor eigenvectors (i.e., the eigenvectors associated with the largest and smallest eigenvalues, respectively). Let:

(6) $$\begin{array}{c} W_{CSP}=\left[w_{1},\ldots ,w_{d}\right]\in R^{D\times d} \end{array}$$

be the matrix that collects these d≤D top (i.e., most discriminating) spatial filters. Given a data matrix $X\in R^{D\times T}$ of observations, all of the same class, the outputs of the spatial filters are defined as:

(7) $$\begin{array}{c} y_{i}=w_{i}^{⊤}X,\;i=1,\ldots ,d,\;\left(d\leq D\right) \end{array}$$

which can be gathered at the d×T output matrix $Y=W_{CSP}^{⊤}X$. Denoting by Σ the sample covariance matrix of X, it follows that the covariance matrix of the outputs is given by $W_{CSP}^{⊤}\Sigma \;W_{CSP}$, while the variance of the output of the *i*-th spatial filter is equal to $w_{i}^{⊤}\Sigma w_{i}$. Finally, not the sample variances, but the log transformed sample variances of the outputs, i.e.,

(8) $$\begin{array}{c} F_{i}=\log\left(w_{i}^{⊤}\Sigma w_{i}\right),\;i=1,\ldots ,d, \end{array}$$

are used as features for the classification of the imagined movements. Observe that, as long as d<D, the dimensionality of the data is reduced.

CSP admits an interesting neurological interpretation. First note that the scalp EEG electrodes measure the addition of numerous sources of neural activity, which are spread over large areas of the neocortical surface, and this does not always allow a reliable localization of the cortical generators of the electrical potentials. It has been suggested that CSP linearly combines the EEG signals so that the sources of interest are enhanced while the others are suppressed [34].

Another interpretation of (4) may be as follows: the basic theory of principal component analysis (PCA) states that maximizing $w^{⊤}\Sigma_{i}w$ finds the direction vector that best fits, in the least-squares sense, the data of class *i* in the *D*-dimensional space. Similarly, minimizing this ratio obtains the opposite effect. Thus, we can interpret that CSP seeks directions that fit well with the data in one class, but are not representative of the data in the other class. By projecting the EEG data onto them, a significant reduction of the variance of one of the classes, while preserving the information content of the other, can be thus obtained.

An interesting generative model perspective has been proposed in [35,36]. Here, the above data matrices are assumed to be generated by a latent variable model:

$$X_{i}\left(:,k\right)=A\;Y_{i}\left(k\right)+N_{i}\left(k\right),$$

where we have used the notation $X_{i}\left(:,k\right)\in R^{D}$ for the *k*-th column of the data matrix $X_{i}$, i.e., it is the observation vector at time *k* for class *i*, i=1,2; $A\in R^{D\times s}$ is a mixing matrix, the same for both classes; $Y_{i}\left(k\right)∼N\left(0,\Gamma_{i}\right)$ is an *s*-dimensional column vector of latent variables (*s* has to be estimated from the data) and $N_{i}\left(k\right)∼N\left(0,\Delta_{i}\right)$ is a *D*-dimensional vector of noise, independent of the data. Here, the covariance matrices $\Gamma_{i}$ and $\Delta_{i}$ are assumed to be diagonal matrices, implying that the latent factors are also independent of each other. Under this model, the columns of matrix A can be regarded as the “spatial patterns” that explain how the EEG data are formed at each electrode location, where the latent variables represent the degree to which each “spatial pattern” appears in the data. Under the assumptions that the noise is negligible and matrix A is square, it is noteworthy that the CSP spatial filters are precisely the columns of the matrix $A^{-⊤}$ [35].

## 3. Divergence-Based Criteria

CSP produces quite good results in general, but also suffers from various shortcomings: e.g., it is sensitive to artifacts [37,38] and its performance is degraded for non-stationary data [39]. For these reasons, CSP is still an active line of research, and a number of variants have been proposed in the literature. In particular, in this paper, we are interested in reviewing CSP-variants based on an information-theoretic framework.

There is a common assumption in the literature that the classes can be modeled by multivariate Gaussian distributions with zero-means and different covariance matrices. This assumption is based on the principle of maximum entropy, not in actual measures of EEG data. By projecting the data onto the principal generalized eigenvectors, CSP transforms them onto a lower dimensional space where the variance of Class 1 is maximized, while the variance for Class 2 is minimized. Conversely, the projection onto the minor generalized eigenvectors has the opposite effect. Since a zero-mean univariate normal variable is completely determined by its variance, we can understand the ratio (4) as a measure of how much the distributions of the projected classes differ from each other (the larger the ratio between the variances, the more different the distributions). By accepting this viewpoint, it is interesting to investigate the ability of other measures of dissimilarity between statistical distributions, rather than the ratio of the corresponding variances, to help in discriminating between the classes. In fact, the most interesting features for classification often belong to those subspaces where there is a large dissimilarity between the conditional densities of the considered classes, which is another justification for proposing a divergence maximization framework in the context of MI-BCI.

In the following sections, we review the main information-theoretic-based approaches.

### 3.1. Criterion Based on the Symmetric Kullback–Leibler Divergence

Divergences are functions that measure the dissimilarity or separation between two statistical distributions. Given two univariate Gaussian densities $N_{1}\left(0,\sigma_{1}\right)$ and $N_{2}\left(0,\sigma_{2}\right)$, their Kullback–Leibler divergence (the KL divergence between two distributions $f_{1}$ and $f_{2}$ is defined as $Div_{KL}\left(f_{1}∥f_{2}\right)=\int_{-\infty }^{\infty }f_{1}\left(x\right)\log\frac{f_{1}\left(x\right)}{f_{2}\left(x\right)}dx$) is easily found to be:

$$Div_{KL}\left(N_{1}\left(0,\sigma_{1}\right)∥N_{2}\left(0,\sigma_{2}\right)\right)=\frac{1}{2}\left(\log\frac{\sigma_{2}^{2}}{\sigma_{1}^{2}}+\frac{\sigma_{1}^{2}}{\sigma_{2}^{2}}-1\right).$$

If the densities have interchangeable roles, it is reasonable to consider the use of a symmetrized measure like the one provided by the symmetrized Kullback–Leibler (sKL) divergence. This is defined simply as:

(9) $$\begin{array}{cc} sDiv_{KL}\left(N_{1}∥N_{2}\right) & =Div_{KL}\left(N_{1}∥N_{2}\right)+Div_{KL}\left(N_{2}∥N_{1}\right)\\ & =\frac{1}{2}\left(\frac{\sigma_{1}^{2}}{\sigma_{2}^{2}}+\frac{\sigma_{2}^{2}}{\sigma_{1}^{2}}\right)-1. \end{array}$$

The resemblance to the CSP criterion (4) is quite obvious, as was already noted, e.g., in [24]. In particular, note that, since $z+\frac{1}{z}$ increases when *z* goes to either infinity or zero, (9) is maximized by either maximizing or minimizing the ratio of the variances $\sigma_{1}$ and $\sigma_{2}$.

The generalization to multivariate data is straightforward. Let $Y=W^{⊤}X$, where X is the observed data matrix and $W=\left[w_{1},\ldots ,w_{d}\right]\in R^{D\times d}$ denotes an arbitrary matrix of spatial filters with 1≤d≤D. Under the assumption that the EEG data are conditionally Gaussian distributed for each class $c_{k}\in \left\{1,2\right\}$, i.e., $X|c_{k}∼N\left(0,\Sigma_{k}\right)$, the spatially-filtered data are also from a normal distribution, i.e., $Y|c_{k}∼N\left(0,{\bar{\Sigma }}_{k}\right)$, where:

$${\bar{\Sigma }}_{k}=W^{⊤}\Sigma_{k}W\in R^{d\times d},$$

k=1,2. The KL divergence between two *d*-dimensional multivariate Gaussian densities $f_{1}=N_{1}\left(0,{\bar{\Sigma }}_{1}\right)$ and $f_{2}=N_{2}\left(0,{\bar{\Sigma }}_{2}\right)$, that is,

$$Div_{KL}\left(f_{1}∥f_{2}\right)=\int N_{1}\left(0,{\bar{\Sigma }}_{1}\right)\log\frac{N_{1}\left(0,{\bar{\Sigma }}_{1}\right)}{N_{2}\left(0,{\bar{\Sigma }}_{2}\right)}dy,$$

can be shown to be (after some algebra):

(10) $$\begin{array}{c} Div_{KL}\left(f_{1}∥f_{2}\right)=\frac{1}{2}\left[\log\frac{|{\bar{\Sigma }}_{2}|}{|{\bar{\Sigma }}_{1}|}-d+\mathrm{trace}{\left({\bar{\Sigma }}_{2}\right)}^{-1}\left({\bar{\Sigma }}_{1}\right)\right], \end{array}$$

where |·| stands for “determinant”. The symmetrized Kullback–Leibler (sKL) divergence between the probability distributions of the two classes is now defined as:

$$\begin{array}{ccc} Div_{sKL}\left(f_{1}∥f_{2}\right) & = & Div_{KL}\left(f_{1}∥f_{2}\right)+Div_{KL}\left(f_{2}∥f_{1}\right)\\ & = & \frac{1}{2}\mathrm{trace}\left({\left(W^{⊤}\Sigma_{1}W\right)}^{-1}\left(W^{⊤}\Sigma_{2}W\right)\right.\\ & & \left.+{\left(W^{⊤}\Sigma_{2}W\right)}^{-1}\left(W^{⊤}\Sigma_{1}W\right)\right)-d, \end{array}$$

where we show again the explicit dependency on W.

We can naturally extend this formula to define the equivalent sKL matrix divergence:

(11) $$\begin{array}{ccc} D_{sKL}\left(W^{⊤}\Sigma_{1}W∥W^{⊤}\Sigma_{2}W\right) & = & \frac{1}{2}\mathrm{trace}\left({\left(W^{⊤}\Sigma_{1}W\right)}^{-1}\left(W^{⊤}\Sigma_{2}W\right)\right.\\ & & \left.+{\left(W^{⊤}\Sigma_{2}W\right)}^{-1}\left(W^{⊤}\Sigma_{1}W\right)\right)-d. \end{array}$$

It has been shown in [23] that the subspace of the filters that maximize the sKL matrix divergence,

(12) $$\begin{array}{cc} W_{sKL} & =\arg\max_{W}D_{sKL}\left(W^{⊤}\Sigma_{1}W∥W^{⊤}\Sigma_{2}W\right), \end{array}$$

coincides with the subspace of those that maximize the CSP criterion, in the sense that the columns of $W_{sKL}$ and $W_{CSP}$ span the same subspace:

(13) $$\begin{array}{ccc} \mathrm{span}(W_{sKL}) & = & \mathrm{span}(W_{CSP}), \end{array}$$

that is, every column of $W_{sKL}$ is a combination of the top spatial filters of $W_{CSP}$ and vice versa.

In practice, $W_{sKL}$ is first used to project the data onto a lower dimensional subspace, and then, $W_{CSP}$ is determined by applying CSP to the projected data. Some advantage can be gained, compared to using CSP only, if in the first step the optimization of the sKL matrix divergence is also combined with some suitable regularization scheme. For example, to fight against issues caused by the non-stationarity of the EEG data, it has been proposed to maximize the regularized objective function [23]:

(14) $$\begin{array}{c} L_{sKL}\left(W\right)=\left(1-\phi \right)D_{sKL}\left(W^{⊤}\Sigma_{1}W∥W^{⊤}\Sigma_{2}W\right)-\phi \Delta \left(W\right), \end{array}$$

where 0≤ϕ<1 and:

(15) $$\begin{array}{c} \Delta \left(W\right)=\frac{1}{2L}\sum_{i=1}^{2}\sum_{k=1}^{L}Div_{KL}\left(N\left(0,W^{⊤}\Sigma_{i,k}W\right)∥N\left(0,W^{⊤}\Sigma_{i}W\right)\right) \end{array}$$

is a regularization term, where we have assumed that *L* trials per class have been performed and $\Sigma_{c,k}$ is the covariance matrix in the *k*-th trial of class c∈{1,2}. This proposed regularization term enforces the transformed data in all the trials to have the same statistical distribution. Other ideas have been proposed in [23], and a related approach can be found in [40]. Observe also that (15) is defined on the basis of the KL divergence, not on its symmetrized version. The KL divergence is calculated by a formula similar to (10), giving:

(16) $$Div_{KL}\left(N\left(0,W^{⊤}\Sigma_{i,k}W\right)∥N\left(0,W^{⊤}\Sigma_{i}W\right)\right)=\frac{1}{2}\left[\log\frac{|W^{⊤}\Sigma_{i}W|}{|W^{⊤}\Sigma_{i,k}W|}-d+\mathrm{trace}{\left(W^{⊤}\Sigma_{i}W\right)}^{-1}\left(W^{⊤}\Sigma_{i,k}W\right)\right].$$

The inverse of $\Sigma_{i,k}$ does not appear in (16), which makes sense if this matrix is ill-conditioned due to insufficient sample size. For this reason, the KL divergence is preferred to its symmetric counterpart. In addition, the logarithm in (16) downweights the effect of $|W^{⊤}\Sigma_{i,k}{W|}^{-1}$ in case $\Sigma_{i,k}$ is nearly singular.

### 3.2. Criterion Based on the Beta Divergence

The beta divergence, which is a generalization of the Kullback–Leibler’s, seems to be an obvious alternative measure of discrepancy between Gaussians. Given two zero-mean multivariate probability density functions $f_{1}\left(y\right)$ and $f_{2}\left(y\right)$, the beta divergence is defined for β>0 as:

$$Div_{\beta }\left(f_{1}\left(y\right)∥f_{2}\left(y\right)\right)=\frac{1}{\beta }\int \left(f_{1}^{\beta }\left(y\right)-f_{2}^{\beta }\left(y\right)\right)f_{1}\left(y\right)dy-\frac{1}{\beta +1}\int \left(f_{1}^{\beta +1}\left(y\right)-f_{2}^{\beta +1}\left(y\right)\right)dy.$$

As $\lim_{\beta \to 0}\frac{f_{1}^{\beta }-f_{2}^{\beta }}{\beta }=\log\left(\frac{f_{1}}{f_{2}}\right)$, it can be shown that the beta divergence converges to the KL divergence for β→0.

Let $f_{1}=N\left(0,{\bar{\Sigma }}_{1}\right)$ and $f_{2}=N\left(0,{\bar{\Sigma }}_{2}\right)$, with ${\bar{\Sigma }}_{i}=W^{⊤}\Sigma_{i}W\in R^{d\times d}$, i=1,2, be the zero-mean Gaussian distributions of the spatially-filtered data. In this case, the symmetric beta divergence between them yields the following closed form formula [41]:

(17) $$D_{s\beta }\left(W^{⊤}\Sigma_{1}W∥W^{⊤}\Sigma_{2}W\right)=\gamma \left(|{\bar{\Sigma }}_{1}{|}^{-\beta /2}+{\left|{\bar{\Sigma }}_{2}\right|}^{-\beta /2}-{\left(\beta +1\right)}^{d/2}\left(\frac{|{\bar{\Sigma }}_{2}{|}^{\frac{1-\beta }{2}}}{|\beta {\bar{\Sigma }}_{1}+{\bar{\Sigma }}_{2}{|}^{1/2}}+\frac{|{\bar{\Sigma }}_{1}{|}^{\frac{1-\beta }{2}}}{|\beta {\bar{\Sigma }}_{2}+{\bar{\Sigma }}_{1}{|}^{1/2}}\right)\right),$$

where $\gamma =\frac{1}{\beta }\sqrt{\frac{1}{{\left(2\pi \right)}^{\beta d}{\left(\beta +1\right)}^{d}}}$. Observe that $D_{s\beta }$ is somewhat protected against possible large increases in the elements of $\Sigma_{1}$ or $\Sigma_{2}$ caused by outliers or estimation errors. For example, if $\Sigma_{i}$ (resp. ${\bar{\Sigma }}_{i}$) grows, i∈{1,2}, then the contribution of all the terms containing $\Sigma_{i}$ (resp. ${\bar{\Sigma }}_{i}$) in (17) tends to vanish. Compared with the previous case, if $\Sigma_{1}$ (for example) increases, then the term:

$$\mathrm{trace}\left({\left(W^{⊤}\Sigma_{2}W\right)}^{-1}\left(W^{⊤}\Sigma_{1}W\right)\right)$$

may dominate (11).

With the necessary changes of divergences being made, the regularizing framework previously defined by Equations (14) and (15) can be easily adapted to the present case [23]. It has been argued in [23] that small values of β penalize abrupt changes in the covariance matrices caused by single extreme events, such as artifacts, whereas a large β is more suitable to penalize the gradual changes over the dataset from trial to trial.

Alternatively, supposing that *L* trials per class are performed, it has been also proposed in [41] to use as the objective function the sum of trial-wise divergences:

$${\bar{D}}_{s\beta }\left(W\right)=\sum_{i=1}^{L}D_{s\beta }\left(W^{⊤}\Sigma_{1,i}W∥W^{⊤}\Sigma_{2,i}W\right),$$

where $\Sigma_{1,i}$ and $\Sigma_{2,i}$ are the covariance matrices in the *i*-th trial of Class 1 and Class 2, respectively.

### 3.3. Criterion Based on the Alpha-Beta Log-Det Divergence

Given the covariance matrices of each class, $\Sigma_{1}$ and $\Sigma_{2}$, an extension of the Kullback–Leibler symmetric matrix divergence given in Equation (11) is the Alpha-Beta log-det (AB-LD) divergence, defined as [42,43]:

(18) $$\begin{array}{ccc} D_{LD}^{\left(\alpha ,\beta \right)}\left(\Sigma_{1}∥\Sigma_{2}\right) & = & \frac{1}{\alpha \beta }\log{\left|\frac{\alpha {\left(\Sigma_{2}^{-\frac{1}{2}}\Sigma_{1}\Sigma_{2}^{-\frac{1}{2}}\right)}^{\beta }+\beta {\left(\Sigma_{2}^{-\frac{1}{2}}\Sigma_{1}\Sigma_{2}^{-\frac{1}{2}}\right)}^{-\alpha }}{\alpha +\beta }\right|}_{+}\\ & & \;\mathrm{for}\;\alpha \neq 0,\;\;\beta \neq 0,\;\;\;\alpha +\beta \neq 0, \end{array}$$

where:

$${\left|x\right|}_{+}=\left\{\begin{array}{cc} x & x\geq 0,\\ 0, & x<0, \end{array}\right.$$

denotes the non-negative truncation operator. For the singular cases, the definition becomes:

(19) $$\begin{array}{ccc} D_{LD}^{\left(\alpha ,\beta \right)}\left(\Sigma_{1}∥\Sigma_{2}\right)\;\;\; & = & \;\;\;\left\{\begin{array}{cc} \frac{1}{\alpha^{2}}\left[\mathrm{tr}\left({\left(\Sigma_{2}^{\frac{1}{2}}\Sigma_{1}^{-1}\Sigma_{2}^{\frac{1}{2}}\right)}^{\alpha }-I\right)-\alpha \log\left|\Sigma_{2}^{\frac{1}{2}}\Sigma_{1}^{-1}\Sigma_{2}^{\frac{1}{2}}\right|\right] & \;\mathrm{for}\;\;\;\alpha \neq 0,\;\beta =0,\\ \frac{1}{\beta^{2}}\left[\mathrm{tr}\left({\left(\Sigma_{2}^{-\frac{1}{2}}\Sigma_{1}\Sigma_{2}^{-\frac{1}{2}}\right)}^{\beta }-I\right)-\beta \log\left|\Sigma_{2}^{-\frac{1}{2}}\Sigma_{1}\Sigma_{2}^{-\frac{1}{2}}\right|\right] & \;\mathrm{for}\;\;\;\alpha =0,\;\beta \neq 0,\\ \frac{1}{\alpha^{2}}\log{\left|{\left(\Sigma_{2}^{-\frac{1}{2}}\Sigma_{1}\Sigma_{2}^{-\frac{1}{2}}\right)}^{\alpha }\left(I+\log{\left(\Sigma_{2}^{-\frac{1}{2}}\Sigma_{1}\Sigma_{2}^{-\frac{1}{2}}\right)}^{-\alpha }\right)\right|}_{+} & \;\mathrm{for}\;\alpha =-\beta ,\\ \frac{1}{2}||\log\left(\Sigma_{2}^{\frac{1}{2}}\Sigma_{1}^{-1}\Sigma_{2}^{\frac{1}{2}}\right){\left|\right|}_{F}^{2} & \;\mathrm{for}\;\;\;\alpha ,\;\beta =0. \end{array}\right. \end{array}$$

**Figure 2:**
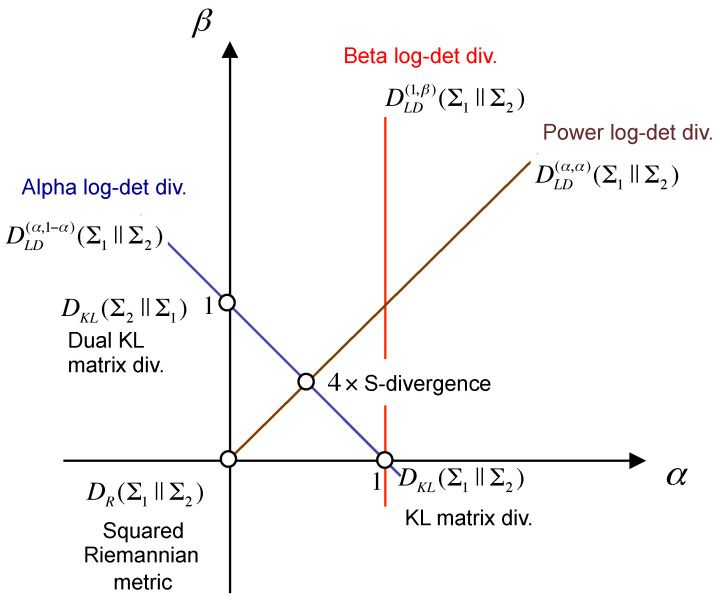
Illustration of the Alpha-Beta log-det divergence (AB-LD) divergence $D_{LD}^{\left(\alpha ,\beta \right)}\left(\Sigma_{1}∥\Sigma_{2}\right)$ in the (α,β)-plane. Note that the position of each divergence is specified by the value of the hyperparameters (α,β). This parameterization smoothly connects several positive definite matrix divergences, such as the squared Riemannian metric (α=0,β=0), the KL matrix divergence or Stein’s loss (α=1,β=0), the dual KL matrix divergence (α=0,β=1) and the *S*-divergence ($\alpha =\frac{1}{2},\beta =\frac{1}{2}$), among others.

It can be easily checked that $D_{LD}^{\left(\alpha ,\beta \right)}\left(\Sigma_{1}∥\Sigma_{2}\right)=0$ iff $\Sigma_{1}=\Sigma_{2}$. The interest in the AB-LD divergence is motivated by the fact that, as can be observed in Figure 2, it generalizes several existing log-det matrix divergences, such as the Stein’s loss (the Kullback–Leibler matrix divergence), the *S*-divergence, the Alpha and Beta log-det families of divergences and the geodesic distance between covariance matrices (the squared Riemannian metric), among others [43].

There is a close relationship between the AB-LD divergence criterion and CSP: it has been shown [42] that the sequence of Courant-like minimax divergence optimization problems [42]

(20) $$w_{\pi_{i}}=\arg\min_{\dim\{W\}=D-i+1}\;\max_{w\in \{W\}}\;D_{LD}^{\left(\alpha ,\beta \right)}\left(w^{⊤}\Sigma_{1}w\;∥\;w^{⊤}\Sigma_{2}w\right),\;i=1,\ldots ,D,$$

yields spatial filters $w_{\pi_{i}}$ that essentially coincide (i.e., up to a permutation $\pi_{i}$ in the order) with the CSP spatial filters $w_{i}$, i.e., with the generalized eigenvectors defined by (5). The permutation ambiguity can be actually avoided if we introduce a suitable scaling $\kappa \in R^{+}$ in one of the arguments of the divergence, so (20) becomes

(21) $$w_{i}=\arg\min_{\dim\{W\}=D-i+1}\;\max_{w\in \{W\}}\;D_{LD}^{\left(\alpha ,\beta \right)}\left(w^{⊤}\Sigma_{1}w\;∥\;\kappa \;w^{⊤}\Sigma_{2}w\right),\;i=1,\ldots ,D,$$

where κ is typically close to the unity.

For $W=\left[w_{1},\ldots ,w_{d}\right]\in R^{D\times d}$ with 1≤d≤D, a criterion based on the AB-LD divergence takes the following form [42]

(22) $$L_{LD}\left(W\right)=D_{LD}^{\left(\alpha ,\beta \right)}\left(W^{⊤}\Sigma_{1}W∥\;\kappa \;W^{⊤}\Sigma_{2}W\right)-\eta \left(P\left(c_{1}\right)R_{1}+P\left(c_{2}\right)R_{2}\right)\;,$$

where $P(c_{1})$ and $P(c_{2})$ are the prior probabilities of Class 1 and Class 2,

(23) $$\begin{array}{cc} R_{1} & =\frac{1}{L}\sum_{i=1}^{L}D_{LD}^{\left(\alpha ,\beta \right)}\left(W^{⊤}\Sigma_{1,i}W∥W^{⊤}\Sigma_{1}W\right), \end{array}$$

(24) $$\begin{array}{cc} R_{2} & =\frac{1}{L}\sum_{i=1}^{L}D_{LD}^{\left(\alpha ,\beta \right)}\left(W^{⊤}\Sigma_{2,i}W∥W^{⊤}\Sigma_{2}W\right), \end{array}$$

where *L* is the number of trials per class and $\Sigma_{1,i}$ and $\Sigma_{2,i}$ are the covariance matrices in the *i*-th trial of Class 1 and Class 2, respectively.

The regularization term:

$$P\left(c_{1}\right)R_{1}+P\left(c_{2}\right)R_{2}$$

may be interpreted as a sort of within-class scatter measure, which is reminiscent of that used in Fisher’s linear discriminant analysis. The parameter η thus controls the balance between the maximization of the between-class scatter and the minimization of the within-class scatter. Observe that when both classes are equiprobable, $P\left(c_{1}\right)=P\left(c_{2}\right)=1/2$, this regularization term is the equivalent of the one defined in Equation (15).

### 3.4. Algorithms for Maximizing the Divergence-Based Criteria

**Figure 3:**
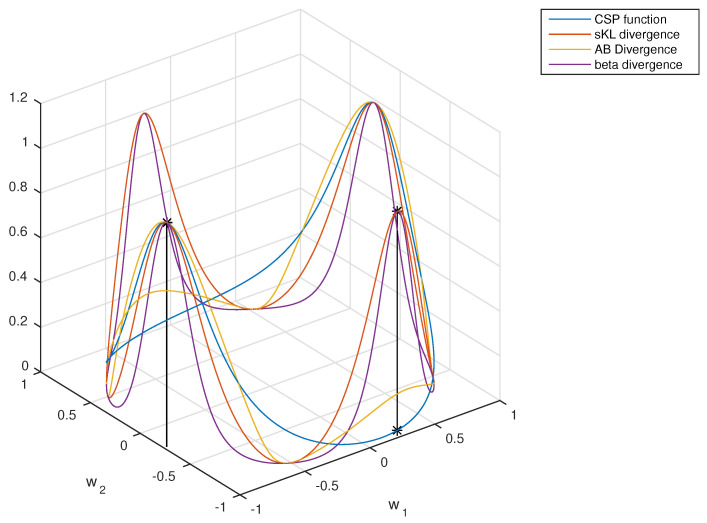
This figure shows the evolution of the common spatial patterns (CSP) criterion function (in blue line), the symmetrized Kullback–Leibler divergence (sKL) (in red line), the symmetrized beta divergence (in purple line) and the AB-LD divergence (in yellow line), all of them as a function of the components of the spatial filter $w=[w_{1},w_{2}]$ in the two-dimensional case, where it is assumed that ${∥w∥}_{2}^{2}=w_{1}^{2}+w_{2}^{2}=1$. All the divergences are normalized with respect to their maximum values, and no regularization has been applied. Observe the coincidence of all the critical points. The covariance matrices were generated at random in this experiment.

To give some idea of how the objective functions are, Figure 3 depicts the divergences defined in Section 3.1, Section 3.2 and Section 3.3 assuming two-dimensional data in the particular case d=1 (so that the projected data are one dimensional). These divergence-based criteria can be optimized in several ways. In practice, a two-step procedure seems convenient, in which a first “whitening” of the observed EEG data is followed by maximization where the search space is the set of the orthogonal matrices.

The rationale is as follows. Observe first that the CSP filters, i.e., the solutions to Equation (5), which is rewritten next for the reader’s convenience,

$$\Sigma_{1}w=\lambda \Sigma_{2}w\to \Sigma_{2}^{-1}\Sigma_{1}w=\lambda w,$$

are also the eigenvectors of the matrix $\Sigma_{2}^{-1}\;\Sigma_{1}$. Since this matrix is not necessarily symmetric, it follows that these eigenvectors do not form an orthogonal set. A well-posed problem can be obtained by transforming the covariance matrices $\Sigma_{i}$ into ${\hat{\Sigma }}_{i}\equiv P\Sigma_{i}P^{⊤}$, where $P\in R^{D}$ is chosen in such a way to ensure the whitening of the sum of the expected sample observations, i.e.,

$$P\left(\Sigma_{1}+\Sigma_{2}\right)P^{⊤}=I.$$

Let w be the matrix that contains the eigenvectors of $\Sigma_{2}^{-1}\;\Sigma_{1}$ in its columns, and let V be the matrix with the eigenvectors of ${\hat{\Sigma }}_{2}^{-1}\;{\hat{\Sigma }}_{1}$. It can be shown that matrix V is orthogonal. Furthermore,

$$W=P^{⊤}V\Lambda \to W^{⊤}=\Lambda^{⊤}V^{⊤}P,$$

where Λ is a diagonal matrix (up to elementary column operations) that contains scale factors. In practice, since only the directions of the spatial filters (i.e., not the magnitude) are of interest, we can ignore the above-defined scale matrix Λ. Then, when only d≤D filters are retained, it can be assumed that $W^{⊤}$ can be decomposed into two components $W^{⊤}=\tilde{R}P$ that successively transform the observations. The first matrix $P\in R^{D}$ is chosen in such a way to ensure the whitening of the sum of the expected sample observations, i.e., $P\left(\Sigma_{1}+\Sigma_{2}\right)P^{⊤}=I$, as was previously explained. The second transformation $\tilde{R}\in R^{d\times D}$ is performed by a semi-orthogonal projection matrix, which rotates and reflects the whitened observations and projects this result onto a reduced *d*-dimensional subspace. This is better seen through the decomposition $\tilde{R}=I_{d}R$, where R is a full rank orthogonal matrix ($RR^{⊤}=I$) and $I_{d}\in R^{d\times D}$ is the identity matrix truncated to have only the first *d* rows.

Let D(·||·) denote any of the previously-studied divergences. The above discussion suggests maximizing the criterion:

(25) $$\begin{array}{ccc} D(W^{⊤}\Sigma_{1}W∥W^{⊤}\Sigma_{2}W) & = & D(\tilde{R}{\tilde{\Sigma }}_{1}{\tilde{R}}^{⊤}∥\tilde{R}{\tilde{\Sigma }}_{2}{\tilde{R}}^{⊤})\\ & = & D(I_{d}R{\tilde{\Sigma }}_{1}R^{⊤}I_{d}∥I_{d}R{\tilde{\Sigma }}_{2}R^{⊤}I_{d})\\ & \equiv & J(R) \end{array}$$

under the constraint that R is an orthogonal matrix, where ${\tilde{\Sigma }}_{i}=P\Sigma_{i}P^{⊤}$.

Now, we face the problem of optimizing J(R) under the orthogonality constraint $RR^{⊤}=I$. This problem can be addressed in several ways, and here, we review two particularly significant approaches.

#### 3.4.1. Tangent Methods

First of all, it has been shown that the gradient of *J* at R on the group of orthogonal matrices is given by [44,45]:

(26) $$\begin{array}{c} \nabla J\left(R\right)=\partial J\left(R\right)-R{\left(\partial J\left(R\right)\right)}^{⊤}R, \end{array}$$

where ∂J(R) is the matrix of partial derivatives of *J* with respect to the elements of R, i.e.,

(27) $$\begin{array}{c} {\left(\partial J\left(R\right)\right)}_{ij}=\frac{\partial J(R)}{\partial r_{ij}}, \end{array}$$

where $r_{ij}$ is the (i,j)th entry of matrix R. Therefore, for steepest ascent search, consider small deviations of R in the direction ∇J(R) as follows:

(28) $$\begin{array}{c} R\to \bar{R}=R+\mu \nabla J\left(R\right), \end{array}$$

with μ>0. If R is orthogonal, this update direction maintains the orthogonality condition, in the sense that $\bar{R}{\bar{R}}^{⊤}=I+o\left(\mu^{2}\right)$. Furthermore, since the first order Taylor expansion of J(R) is:

(29) $$\begin{array}{c} J(R+\Delta R)=J(R)+<\partial J(R)|\Delta R>+o(\Delta R), \end{array}$$

where $<A|B>=\mathrm{trace}\left(A^{⊤}B\right)$ represents the inner product of two matrices, if R is modified into $\bar{R}$, it follows that:

(30) $$\begin{array}{c} J\left(\bar{R}\right)=J\left(R\right)+\mu <\partial J\left(R\right)|\nabla J\left(R\right)>+o\left(\mu \right). \end{array}$$

Some algebra shows that:

(31) $$\begin{array}{ccc} <\partial J(R)|\nabla J(R)> & = & \frac{1}{2}<\nabla J\left(R\right)|\nabla J\left(R\right)> \end{array}$$

which is always positive, and therefore, *J* always increases. The steepest ascent method thus becomes:

(32) $$\begin{array}{c} R_{t+1}=R_{t}+\mu \nabla J\left(R\right)=\left[I+\mu H(R_{t})\right]R_{t}, \end{array}$$

where:

(33) $$\begin{array}{c} H\left(R_{t}\right)=\partial J\left(R_{t}\right)R_{t}^{⊤}-R_{t}\partial J{\left(R_{t}\right)}^{⊤}. \end{array}$$

A drawback of this approach is that, as the algorithm iterates, the orthogonality constraint may be no longer satisfied. One possible solution is to re-impose the constraint from time to time by projecting R back to the constraint surface, which may be performed using an orthogonalization method such as the Gram–Schmidt technique. This approach has been used, e.g., in [42].

#### 3.4.2. Optimization on the Lie Algebra

Alternatively, R can be forced to remain always on the constraint surface using an iteration of the form [44]:

(34) $$\begin{array}{c} R_{t+1}=Q_{t}R_{t}, \end{array}$$

where $Q_{t}=\exp\left(M_{t}\right)$ and $M_{t}$ is skew symmetric, i.e., $M_{t}=-M_{t}^{⊤}$. As the exponential of a skew symmetric matrix is always orthogonal, we ensure that $R_{t+1}$ is orthogonal, as well, supposing $R_{t}$ to be. Technically speaking, the set of the skew symmetric matrices is called a Lie algebra, and the idea is to optimize *J* moving along it. As the update rule for R given in (34) may be also considered as an update for M from the zero matrix to its actual value $M_{t}$, the algorithm is as follows:

1. Start at the zero matrix 0.
2. Move from 0 to
  (35) $$\begin{array}{c} M_{t}=\mu \nabla_{M}{J|}_{M=0}, \end{array}$$
  where $\nabla_{M}J$ is the gradient of *J* with respect to M in the Lie algebra:
  (36) $$\begin{array}{c} \nabla_{M}J=\partial J\left(R\right)R^{⊤}-R\partial J{\left(R\right)}^{⊤}. \end{array}$$
3. Define $Q_{t}=\exp\left(M_{t}\right)$, and use it to come back into the space of the orthogonal matrices.
4. Update $R_{t+1}=Q_{t}R_{t}$.

Note that, for small enough μ, we have that $\exp\left(M\right)=\exp\left(\mu \nabla_{M}J\right)\approx I+\mu \nabla_{M}J$, so that (34) coincides with (32). From this viewpoint, it may seem that (34), which is used in [23,41], is superior to (32), in the sense that includes (32) as a particular case. Nevertheless, the main drawback of (34) is that it is necessary to calculate the exponential of a matrix, which is a somewhat “tricky” operation [46]. In both approaches, the optimal value of μ can be chosen by a line search along the direction of the gradient.

More advanced optimization techniques, like the standard quasi-Newton algorithms based on the Broyden–Fletcher–Goldfarb–Shannon (BFGS) method [24] have been recently extended to work on Riemannian manifolds [47]. The algorithm used in Section 6 for the optimization of the AB-LD divergence criterion [42], which we will denote in this paper as the Sub-LD algorithm, is based on the BFGS implementation on the Stiefel manifold of semi-orthogonal matrices [48]. Finally, note that spatial filters can be computed all at a once, yielding the so-called subspace approach, or one after the other by a sequential procedure, which is called the deflation approach. In the latter case, the problem is repeatedly solved for d=1, and a projection mechanism is used to prevent the algorithms from converging to previously found solutions [23].

#### 3.4.3. Post-Processing

Finally, it has to be pointed out that, by maximizing any divergence, we may not obtain the CSP filters, i.e, the vectors $w_{i}$ computed by the CSP method, but a linear combination of them [23,42]. The filters are actually determined by applying CSP to the projected data in a final step.

## 4. The Information Theoretic Feature Extraction Framework

Information theory can play a key role in the dimensionality reduction step that extracts the relevant subspaces for classification. Inspired by some other papers in machine learning, the authors of [49] adopted an information theoretic feature extraction (ITFE) framework based on the idea of selecting those features, which are maximally informative about the class labels. Let X be the *D*-dimensional random variable describing the observed EEG data. In this way, the desired spatial filters are the ones that maximize the mutual information between the output random variable $Y=w^{⊤}X$ and a class random variable *C* that represents the true intention of the BCI user, i.e.,

(37) $$\begin{array}{ccc} w_{*} & = & \arg\max_{w}I\left(C\;;\;w^{⊤}X\right). \end{array}$$

As was noted in [49], this criterion can be also linked with the minimization of an upper-bound on the probability of classification error. Consider the entropy H(C) and a function:

(38) $$\begin{array}{c} U\left(\gamma \right)=1-2^{-(H(C)-\gamma )}, \end{array}$$

which was used in [50] to obtain an upper-bound for the probability of error:

(39) $$\begin{array}{ccc} P_{e} & \leq & U(I(C\;;\;Y)). \end{array}$$

Since U(γ) is a strictly monotonous descending function, the minimization of the upper-bound of $P_{e}$ is simply obtained through the maximization of the mutual information criterion:

(40) $$\begin{array}{c} J_{ITFE}\left(w\right)=I\left(C\;;\;w^{⊤}X\right). \end{array}$$

Although the samples in each class are assumed to be conditionally Gaussian distributed, the evaluation of this criterion also requires one to obtain $h(w^{⊤}X)$, the differential entropy of the output of the spatial filter, which is non-trivial to evaluate, and therefore, it has to be approximated. The procedure starts by choosing the scale of the filter that normalizes the random variable $w^{⊤}X$ to unit variance. Assuming that $w^{⊤}X$ is nearly Gaussian distributed, the differential entropy of this variable is approximated with the help of a truncated version of the Edgeworth expansion for a symmetric density [51]:

(41) $$\begin{array}{c} h\left(w^{⊤}X\right)\approx h_{g}\left(w^{⊤}X\right)-\frac{1}{48}{\left(k_{4}\left(w^{⊤}X\right)\right)}^{2}, \end{array}$$

where $h_{g}\left(w^{⊤}X\right)$ denotes the entropy of a Gaussian random variable with power $E[|w^{⊤}X{|}^{2}]=1$ and kurtosis $k_{4}\left(w^{⊤}X\right)$. By expressing the value of the kurtosis of a mixture of conditional Gaussian densities in terms of the conditional variances of the output for each class, after substituting these values in (41), the authors of [49] arrive to the approximated mutual information criterion that they propose to maximize:

(42) $$\begin{array}{cc} {\tilde{J}}_{ITFE}\left(w\right) & \equiv -\frac{1}{2}\sum_{k=1}^{n_{c}}P\left(c_{k}\right)\log_{2}\left(w^{⊤}\Sigma_{k}w\right)-\frac{3}{16}{\left(\sum_{k=1}^{n_{c}}P\left(c_{k}\right)\left({\left(w^{⊤}\Sigma_{k}w\right)}^{2}-1\right)\right)}^{2}\\ & \approx J_{ITFE}\left(w\right), \end{array}$$

where $n_{c}$ is the number of classes and $\Sigma_{k}$ denotes the conditional covariance matrix of the *k*-th class.

On the one hand, for only two classes ($n_{c}=2$), the exact solution of the ITFE criterion can be shown to coincide with the one of CSP. On the other hand, for multiclass scenarios ($n_{c}>2$), it is proposed to use a Joint Approximate Diagonalization (JAD) procedure (which is no longer exact) for obtaining the independent sources of the observations and then retain only those sources that maximize the approximated mutual information with the class labels.

## 5. Non-Information-Theoretic Variants of CSP

In this section we review, for the purposes of comparison, some variants of CSP that are not based on information-theoretic principles. Although CSP is considered to be the most effective algorithm for the discrimination of motor imagery movements, it is also sensitive to outliers. Several approaches have been proposed to improve the robustness of the algorithm.

Using the sample estimates of the covariance matrices, the CSP criterion (4) can be rewritten as:

(43) $$\begin{array}{c} \hat{J}\left(w\right)=\frac{w^{⊤}\Sigma_{1}w}{w^{⊤}\Sigma_{2}w}=\frac{w^{⊤}X_{1}X_{1}^{⊤}w}{w^{⊤}X_{2}X_{2}^{⊤}w}=\frac{∥w^{⊤}X_{1}{∥}_{2}^{2}}{∥w^{⊤}X_{2}{∥}_{2}^{2}}\;, \end{array}$$

where $X_{i}$ denotes the data matrix of class *i*. Therefore, CSP is not a robust criterion as large outliers are favored over small data values by the square in Equation (43). To fix this problem, some approaches use robust techniques for the estimation of the covariance matrices [37]. Alternatively, as presented in [52], a natural extension of CSP that eliminates the square operation, having it replaced by the absolute value, is given by:

(44) $${\hat{J}}_{1}\left(w\right)=\frac{∥w^{⊤}X_{1}{∥}_{1}}{∥w^{⊤}X_{2}{∥}_{1}}.$$

This $l_{1}$-norm-based CSP criterion is more robust against outliers than the original $l_{2}$-norm-based formula (43). However, $l_{1}$-norm CSP does not explicitly consider the effects of other types of noise, such as those caused by ocular movements, eye blinks or muscular activity, supposing that they are not completely removed in the preprocessing step [53,54]. To take them into account, [55] added a penalty term in the denominator of the CSP-$l_{2}$ objective function, obtaining:

(45) $${\hat{J}}_{1r}\left(w\right)=\frac{∥w^{⊤}X_{1}{∥}_{2}^{2}}{∥w^{⊤}X_{2}{∥}_{2}^{2}+\rho R\left(w\right)},$$

where R(w) is some measure of the intraclass scattering of the filtered data in each of the classes, so the maximization of ${\hat{J}}_{1r}\left(w\right)$ encourages the minimization of R(w), and ρ is a positive tuning parameter. Finally, a generalization of the $l_{1}$-norm-based approach has been proposed in [56,57], which explores the use of $l_{p}$ norms through the following criterion:

(46) $${\hat{J}}_{1p}\left(w\right)=\frac{∥w^{⊤}X_{1}{∥}_{p}^{1/p}}{∥w^{⊤}X_{2}{∥}_{p}^{1/p}}.$$

Other approaches for regularizing the original $l_{2}$-norm based CSP algorithm include performing a robust estimation of the covariance matrices $\Sigma_{i}$ or adding a penalty term Δ in the objective function. With regard to the first approach, [58] proposes the use of information from various subjects as a regularization term, so the sample covariance matrices Σ are substituted in the formulas for:

$$\tilde{\Sigma }=\left(1-\psi \right)\Sigma +\psi \frac{1}{\left|S\right|}\sum_{k\in S}\Sigma^{k},$$

where S is a set of subjects whose data have been previously recorded, $\Sigma^{k}$ is the sample covariance matrix of the *k*-th subject and ψ∈(0,1) is a regularization parameter. Related approaches can be found in [21,37,38,59,60,61,62,63]. Finally, in [7] the covariance matrices are estimated using data originating from specific regions of interest within the brain.

The second regularization approach consists of including a penalty term in the CSP objective function [64]. The regularized CSP objective functions can be represented as:

(47) $$\begin{array}{cc} {\tilde{J}}_{1}\left(w\right) & =\frac{w^{⊤}\Sigma_{1}w}{w^{⊤}\Sigma_{2}w+\alpha \Delta \left(w\right)} \end{array}$$

(48) $$\begin{array}{cc} {\tilde{J}}_{2}\left(w\right) & =\frac{w^{⊤}\Sigma_{2}w}{w^{⊤}\Sigma_{1}w+\alpha \Delta \left(w\right)} \end{array}$$

where α is the regularization parameter. The regularized Tikhonov-CSP approach (RTCSP) penalizes the solutions with large weights by using a penalty term Δ(w) of the form:

Δ(w)=∥w∥.

The filters w can computed by solving an eigenvalue problem similar to that of the standard CSP algorithm. Specifically, the stationary points of ${\tilde{J}}_{1}\left(w\right)$ verify [64]:

$${\left(\Sigma_{2}+\alpha I\right)}^{-1}\Sigma_{1}w=\lambda w.$$

Similarly, the stationary points of ${\tilde{J}}_{2}\left(w\right)$ are the eigenvectors of matrix ${\left(\Sigma_{1}+\alpha I\right)}^{-1}\Sigma_{2}$. Observe that it is necessary to optimize both objective functions, as the stationary points of any of them alone maximize the variance of one class, but do not minimize the variance of the other class.

**Figure 4:**
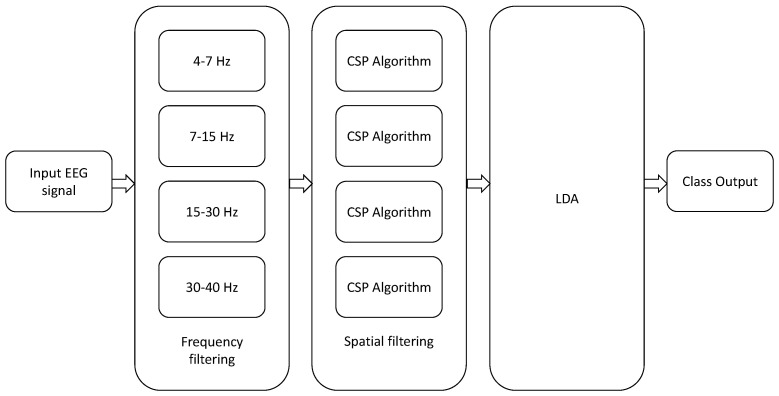
Architecture of filter bank CSP. LDA is shorthand for Linear Discriminant Analysis.

Finally, all the previous approaches admit the following generalization: in traditional CSP, the EEG data is usually band-pass pre-filtered using one single filter between 8 and 30 Hz, which is a range that covers the so-called “alpha”, “beta” and “mu” EEG bands. An straightforward extension, known as the filter bank CSP (FBCSP) technique, was proposed in [30], where the input MI-EEG signals are bandpass filtered between different bands of frequency ((4–8 Hz), (8–12 Hz), *…*, (36–40 Hz)) and the CSP algorithm, or any of its variants, is applied to each band for the computation of the spatial filters. The results of all analyses are then combined to form the final response (see Figure 4). Similar approaches have been proposed in [10,65,66]. An extension to the multiclass problem can be found in [67]. Since the optimal frequency bands can vary from subject to subject, several alternative approaches have been proposed that combine the time-frequency characteristics of the EEG data [68,69] for improving the classification accuracy and reducing the number of electrodes [70].

## 6. Experimental Results

Initially, we will test the algorithms using real datasets obtained from the BCI competition III (dataset 3a) and BCI competition IV (dataset 2a), which are publicly available at [71]. On the one hand, the dataset 3a from BCI competition III consists of EEG data acquired from three subjects (k3b, k6b and l1b) at a sampling frequency of 250 Hz using a 60-channel EEG system. In each trial, an arrow to the left, right, up or down was shown on a display for a few seconds, and in response to the stimulus, the subject was asked to respectively perform left hand, right hand, tongue and foot MI movements. The dataset consists of 90 trials per class for Subject k3b and 60 trials per class for Subjects k6b and l1b. On the other hand, the dataset 2a from BCI competition IV was acquired by using 22 channels from nine subjects (A01–A09) while also performing left hand, right hand, tongue and foot MI movements following a similar procedure. The signals were also sampled at 250 Hz and were recorded in two sessions on different days, each of them with 72 trials per each class.

For a total of four possible motor-imagery (MI) movements, (42)=6 different combinations of pairs of MI movements (i.e., left hand-right hand, left hand-foot, left hand-tongue, right hand-foot, right hand-tongue, foot-tongue) can be formed. The experiments below consider all possible combinations: since 12 users are available and for nine of them we have recordings performed on two different days, this makes a total of 3×6+9×6×2=126 different experiments. We repeated eight times each of these 126 possible experiments, and results were averaged. For each repetition, 60 trials were selected at random from each MI movement, which were split into 40 trials for training and 20 trials for testing. Additionally, in the case of the BCI competition IV, we averaged over the two sessions conducted for each user to avoid biasing the statistical tests. As a result, 3×6+9×6×2/2=72 averaged performance measures are finally available for each algorithm. The data have been initially bandpass filtered between the cut-off frequencies of 8–30 Hz, except before using the FBCSP method, which as we explained in Section 5, considers four bands for covering the frequency range between 4 and 40 Hz. The information of the classes in each trial is summarized by their respective covariance matrices. These matrices are estimated, normalized by their trace and used as input to the algorithms that carry out the calculation of the spatial filters prior to the MI classification, which is performed by using linear discriminant analysis (LDA).

**Figure 5:**
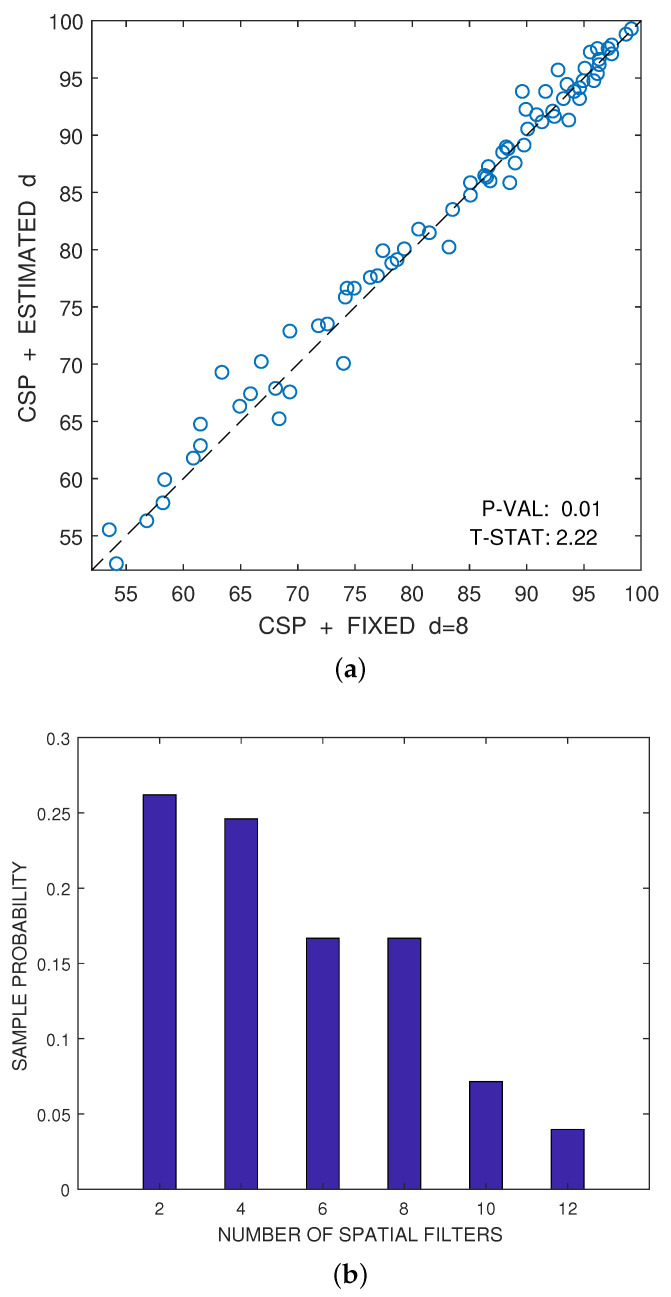
Illustration of the advantages in performance of using an automatic cross-validation method to estimate the best even number of features *d* with respect to using an a priori fixed value of *d*. The automatic method relies on the technique proposed in [72], which was implemented here using one-sided *t*-tests of significance instead of the original two-sided tests. (**a**) Scatter plot comparison of the accuracies (in percentage) obtained by the CSP algorithm for fixed d=8 (*x*-axis) and for the automatic estimation of *d* (*y*-axis); (**b**) histogram of the estimated best even number of features *d*.

The only parameter of the CSP algorithm is the number of spatial filters that one would like to consider. Although, this number *d* is usually fixed a priori for each dataset, it is advantageous to estimate automatically the best number of spatial filters for each user by using the combination of cross-validation and hypothesis testing proposed in [72]. Figure 5a illustrates this fact. The figure represents the scatter plot of the accuracies, expressed as a percentage, that have been respectively obtained by the CSP algorithm for a fixed value of d=8 (*x*-axis) and for the estimation of the best value of *d* (*y*-axis). These estimated accuracies have been obtained by averaging eight test samples, as explained above. The accuracies obtained for different individuals or for different pairs of conditions can be reasonably considered approximately independent and nearly Gaussian. Under this hypothesis, a one-sided paired *t*-test of statistical significance can be used to compare the results obtained by both alternatives. Let $\delta f\left(m\right)=f_{y}\left(m\right)-f_{x}\left(m\right)$ be the paired differences of accuracy ((*y*-axis value) vs. (*x*-axis value)) for m=1,…,M, where M=72 is the number of samples. Then, the averaged difference is:

(49) $$\begin{array}{c} \bar{\Delta f}=\frac{1}{M}\sum_{m=1}^{M}\delta f\left(m\right) \end{array}$$

and the unbiased estimate of its variance is:

(50) $$\begin{array}{c} s^{2}=\frac{s_{\Delta f}^{2}}{M}, \end{array}$$

where $s_{\Delta f}^{2}=\frac{1}{M-1}\sum_{m=1}^{M}{\left(\delta f\left(m\right)-\bar{\Delta f}\right)}^{2}$. Under the null hypothesis (H0) that the expected performance values coincide, i.e., $E\left[f_{y}\left(m\right)\right]=E\left[f_{x}\left(m\right)\right]$, the *t*-statistic:

(51) $$\begin{array}{c} T-STAT=\frac{\bar{\Delta f}}{s_{\Delta f}/\sqrt{M}}. \end{array}$$

follows a Student’s *t* distribution with M−1 degrees of freedom. Thus, the probability that the null hypothesis can generate a *t*-statistic larger than T−STAT gives the *p*-value of the right-sided test:

(52) $$\begin{array}{c} P-VAL=Prob(t>T-STAT|H0). \end{array}$$

The more positive is T−STAT, the smaller is the P−VAL, and the probability of observing a *t*-statistics larger than T−STAT decreases under the null hypothesis. When the *p*-value falls below the 0.05 threshold of significance, the hypothesis of not having a performance improvement when using the alternative procedure can be rejected, because this would correspond to a quite improbable situation. On the contrary, if the *p*-value of the right-sided test is above 0.05, the null hypothesis cannot be rejected.

In this particular case, the *p*-value of the test in Figure 5a is below 0.05; therefore, one can reject the hypothesis that the automatic estimation of *d* does not improve the results over the method that a priori selects d=8 filters.

We briefly name and describe below some of the implementations that optimize the already mentioned criteria for dimensionality reduction in MI-BCIs. Because of the substantially higher computational complexity of most of the alternatives to CSP (see Table 1), it is not practical to develop a specific automatic estimation procedure of the number of spatial filters for each of them. For this reason, we will consider in their implementations the same number of spatial filters that was automatically estimated for CSP.

- CSP (see Section 2) and ITFE (see Section 4): apart from the number of spatial filters, these two methods do not have hyper-parameters to tune. Their respective algorithms have been implemented according to the specifications given in [18,49].
- RTCSP (see Section 5): RTCSP has a regularization parameter, which has been selected by five-fold cross-validation in {0,0.1,0.2,…,1}. The MATLAB implementation of this algorithm has been obtained from [73].
- FBCSP (see Section 5): In this case, we have used a variation of the algorithm in [30]. The selected frequency bands correspond to the brainwaves *theta* (4–7 Hz), *alpha* (8–15 Hz), *beta* (16–31 Hz) and *low gamma* (32–40 Hz), where five-fold cross-validation has been used to select the best combination of these frequency bands. We extract *d* features from each band, where *d* is selected using the method in [72].
- DivCSP (see Section 3.2 and Section 3.4). The values of β and ϕ (the regularization parameter) have been selected by five-fold cross-validation, β∈[0,1], ϕ∈[0,0.5]. This divergence includes the KL divergence as a particular case when β=0. MATLAB code of the algorithm has been downloaded from [74] and used without any modification. Optimization has been performed using the so-called subspace method (see Section 3.4).
- Sub-LD (sub-space log-det): this algorithm, which also belongs to the class of the subspace methods, is based on the criterion in [42] to maximize the Alpha-Beta log-det divergence (see Section 3.3 and Section 3.4). In this paper, the implementation of the algorithm is based on the BFGS method on the Stiefel manifold of semi-orthogonal matrices and takes as the initialization point the solution obtained by the CSP algorithm. The regularization parameter η has been chosen by five-fold cross-validation in the range of values (−0.2,0.2), which are not far from zero. The negative values of η favor the expansion of the clusters, while the positive values favor their contraction. For η close to zero, the solution of this criterion should not be far from that of CSP, which improves the convergence time of the algorithm and reduces the impact of the values of α,β in the results, so both parameters have been fixed to 0.5.

Table 1 shows the typical execution time of a single run of each algorithm, programmed in MATLAB language, in a PC with Intel I7-6700 CPU @ 3.4-GHz processor and 16 GB of RAM. The algorithms that use cross-validation for selecting the hyper-parameters need more iterations, hence the run time has to be multiplied by the number of the hyper-parameters combinations that are evaluated.

**Figure 6:**
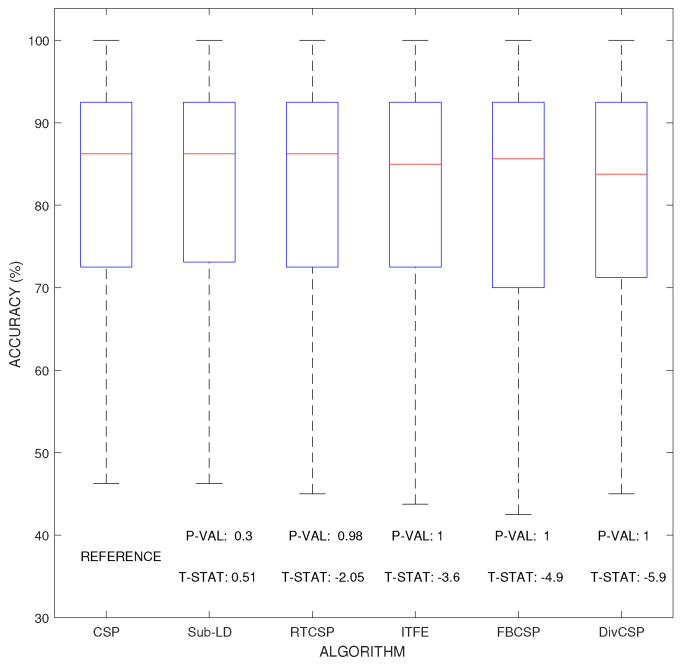
Comparison of the expected accuracy percentages obtained by each of the considered algorithms. The figure shows box-plot illustrations where the median is shown in red line, while the 25% and 75% percentiles are respectively at the bottom and top of each box. Larger positive values T−STAT≫0 and smaller P−VAL≪1/2 would correspond with greater expected improvements over CSP. However, none of the *p*-values, which are shown below their respective box-plots, is able to attain the 5% threshold level of significance (P−VAL<0.05), so the possible improvements cannot be claimed to be statistically significant with respect to those obtained by CSP.

**Figure 7:**
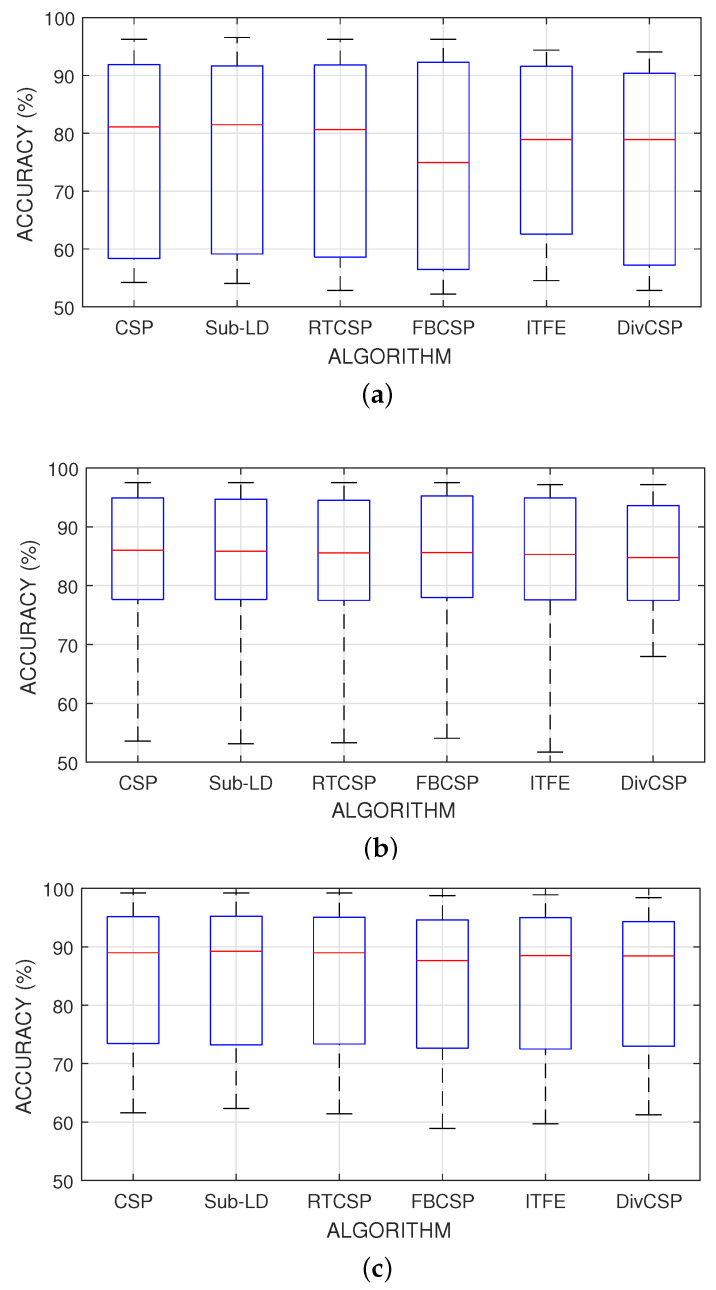
Performance of the algorithms for different motor imagery combinations involving the right hand. (**a**) Right-hand versus left-hand motor imagery classification; (**b**) right-hand versus feet motor imagery classification; (**c**) right-hand versus tongue motor imagery classification.

**Figure 8:**
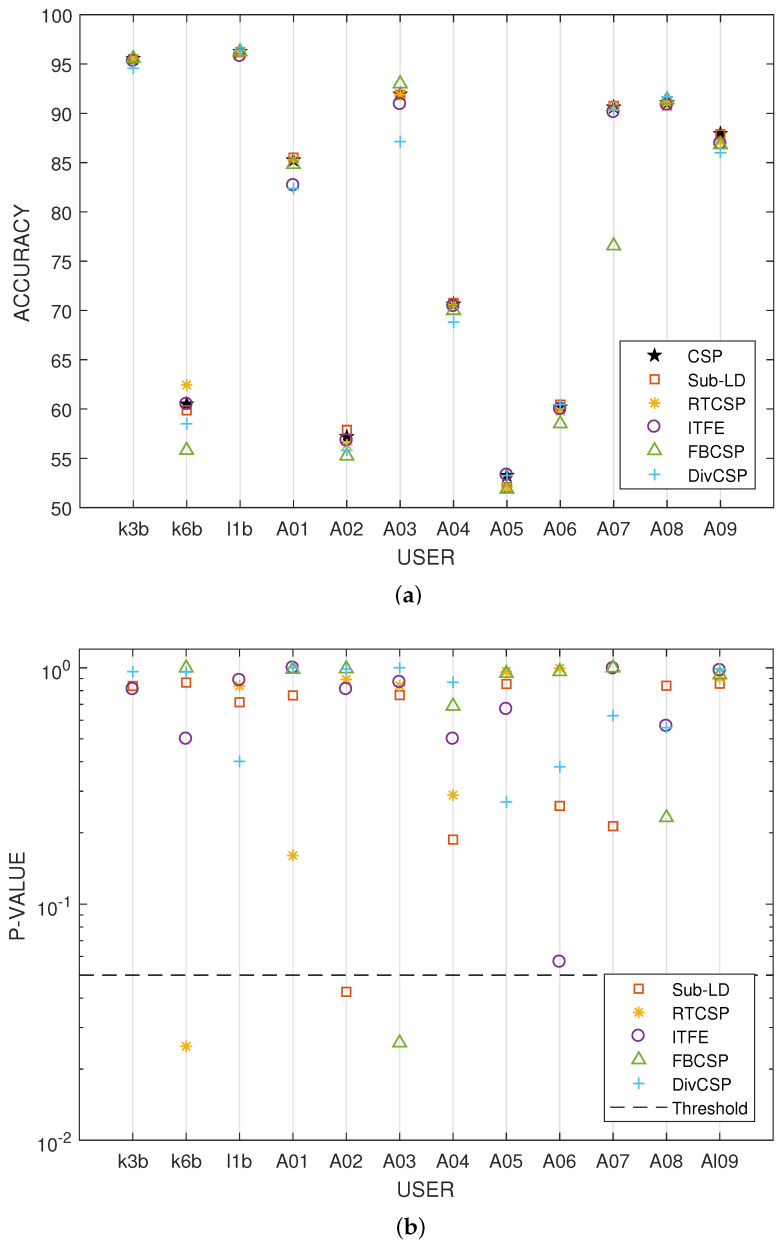
Accuracy percentages and *p*-values for the testing of an improvement in performance over CSP when the right hand versus left hand movement imagination are discriminated. The results reveal that, in general and except in a few isolated cases, the null hypothesis that the other methods do not significantly improve the performance over CSP cannot be discarded. (**a**) Average accuracy obtained by the algorithms for each subject; (**b**) *p*-values of the *t*-tests that compare whether the performance of the alternative algorithms is significantly better than the one obtained by CSP. The horizontal dashed line represents the threshold level of significance of 5%.

Figure 6 represent the boxplot of the accuracy of the algorithms, considering together all the combinations of the motor imagery movements from all subjects in datasets III 3a and IV 2a. The *p*-values and *t*-statistics shown below the box-plots of Figure 6 are above the 5% threshold of significance, revealing that, in this experiment, one cannot reject the null hypotheses. It follows that the expected accuracies of the alternative algorithms are not significantly higher than the expected accuracies obtained with CSP. Supporting this conclusion, Figure 7 represents the specific boxplots that corresponds to MI movements involving the right hand. Additionally, we have tested, in the case “left hand versus right hand”, whether the improvement obtained by using the alternative algorithms is significant or not. The accuracy in the classification and the corresponding *p*-values of the tests are shown in Figure 8. The results reveal that, in general and except in a few isolated cases, the null hypothesis that the other methods do not significantly improve performance over CSP cannot be discarded.

**Figure 9:**
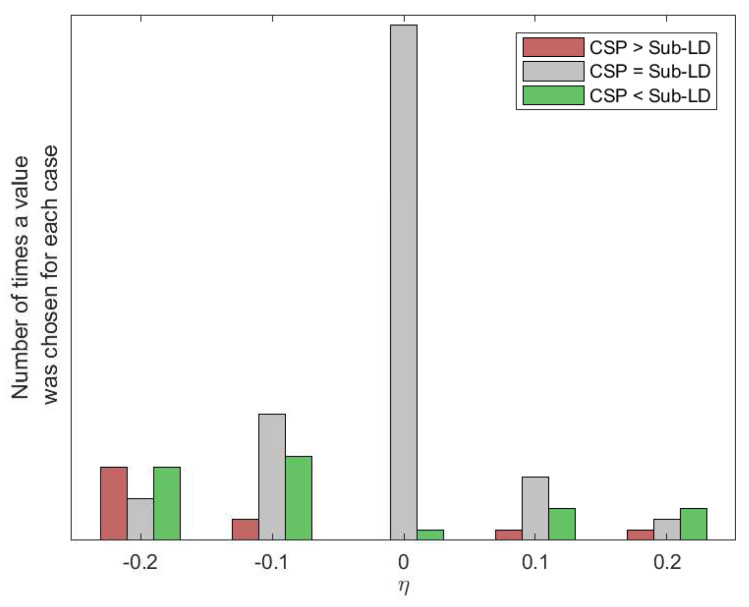
Histogram of the values of the regularization parameter in the Sub-LD algorithm that have been chosen by cross-validation.

**Figure 10:**
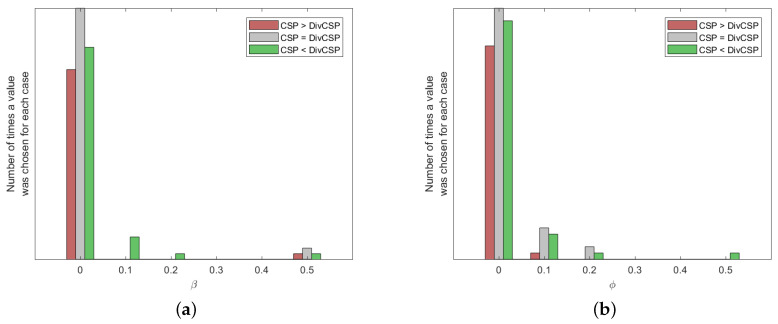
Histogram of the hyper-parameters of the DivCSP algorithm selected by cross-validation. (**a**) Case with β∈[0,0.5] and ϕ=0; (**b**) case with β=0.5 and ϕ∈[0,0.5].

The results of Figure 6 were obtained by choosing through cross-validation the best possible values for the different parameters of the algorithms. Figure 9 and Figure 10 show how many times each value of the parameters has been selected after cross-validation. They also show the number of times that CSP outperformed the corresponding algorithm, the number of times that the algorithm outperformed CSP or the cases in which both of them were equivalent. Without limiting the foregoing, it must be also remarked that the alternative algorithms perform better than CSP for some subjects and MI movements.

### Results on Artificially Perturbed Data

In order to study the performance of the algorithms under artificial perturbations of the datasets we have conducted two experiments. The first one consists of introducing random label changes in the real datasets, while the second one defines sample EEG covariance matrices for each condition and artificially introduces outlier covariance matrices in the training procedure to quantify the resulting deterioration in performance.

**Figure 11:**
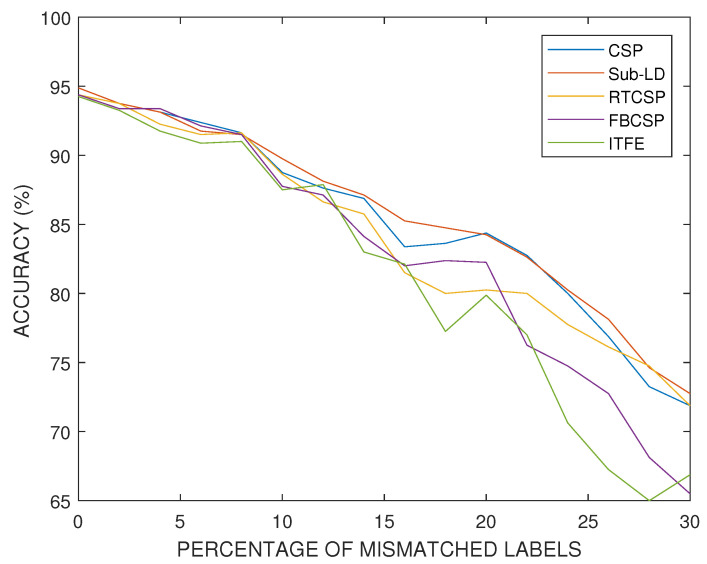
Comparison of the accuracy percentages obtained by each of the considered algorithms with respect to the percentage of mismatched labels in the training set. This experiment illustrates deterioration of the performance of the algorithms with respect to the increase of the percentage of randomly switched labels of the motor imagery movements.

Exchanging labels of the training set at random is one of the most harmful perturbations that one can consider in a real experiment. It models the failure of the subjects to imagine the correct target MI movements due to fatigue or lack of concentration. For this experiment, we selected a subject who has a relatively good performance in absence of perturbations. Figure 11 presents the progressive degradation of the accuracy of the algorithms as the percentage of mismatched labels increases.

In the second experiment, we have created artificial EEG data and consider the effect of adding random outliers. The artificial data were generated starting from two auxiliary covariance matrices $C_{k},\;k=1,2$ for the construction of the conditional covariance matrices of each class. These covariances were generated randomly by drawing two random Gaussian matrices $A^{\left(k\right)}$ with i.i.d. elements $a_{ij}^{\left(i\right)}∼N\left(0,1\right)$ and forming the covariance matrices with $C_{k}=A^{\left(k\right)}{\left(A^{\left(k\right)}\right)}^{⊤},\;k=1,2$. In order to control the difficulty of the classification problem, we introduce a dissimilitude parameter δ∈[0,1] that interpolates between the two auxiliary covariance matrices as follows:

(53) $$\begin{array}{cc} \Sigma_{1} & =C_{1}^{1/2}{\left(C_{1}^{-1/2}C_{2}C_{1}^{-1/2}\right)}^{\frac{\left(1-\delta \right)}{2}}C_{1}^{1/2} \end{array}$$

(54) $$\begin{array}{cc} \Sigma_{2} & =C_{2}^{1/2}{\left(C_{2}^{-1/2}C_{1}C_{2}^{-1/2}\right)}^{\frac{\left(1-\delta \right)}{2}}C_{2}^{1/2} \end{array}$$

In this way, when δ=0, the two interpolated covariance matrices coincide $\Sigma_{1}=\Sigma_{2}$, and it is impossible to distinguish between them. On the contrary, when δ=1, we obtain the original randomly generated matrices $\Sigma_{1}=C_{1}$ and $\Sigma_{2}=C_{2}$. The matrices $\Sigma_{k}$ are used as the expected covariance matrix of the observations for class *k*, while the sample covariance matrices for each trial are generated from a Wishart distribution with scale matrix $\frac{1}{T}\Sigma_{k}$ and *T* degrees of freedom (where *T* denotes the trial length). The outlier matrices have been generated following a similar scheme, though interpolation is not used and the resulting covariances are scaled by a factor of five.

**Figure 12:**
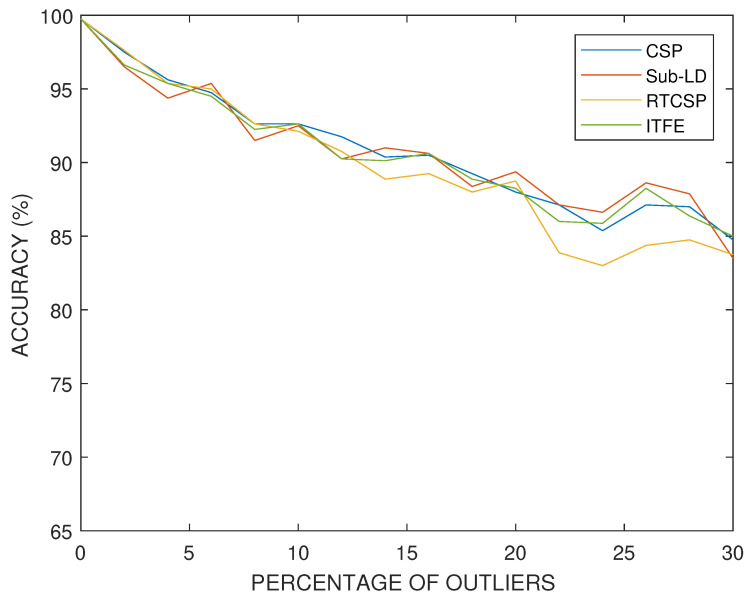
Accuracy percentages versus the percentage of training trials with outliers in a synthetic classification experiment.

In our simulations with artificial data, we have set the dissimilitude parameter to δ=0.1. The results obtained for artificial data and with different percentages of outlier covariance matrices in the training set are shown in Figure 12. One can observe how the performance progressively deteriorates with the number of outliers, similarly for all the methods, although at a smaller rate than in the case having the same percentage of mismatched labels. The parameters of the algorithms have been selected by cross-validation.

## 7. Conclusions

In this paper, we have reviewed several information theoretic approaches for motor-imagery BCI systems. In particular, we have focused on those based on the Kullback–Leibler divergence, Beta divergence, Alpha-Beta log-det divergence and information theoretic feature extraction, exploring the existing links with common spatial patterns, which is a widely-used technique for spatial filtering in BCI applications. The performance of all these methods has been evaluated through experimental simulations using real and synthetic data. In general, the results obtained for real data from BCI competitions reveal a similar performance for all the considered criteria in terms of their percentages of accuracy. However, CSP clearly outperforms the other methods when comparing the required computational burdens. In the case of synthetic data with outliers, a comparison of the divergence-based methods with small regularization parameters reveals that they can slightly increase the frequency of obtaining a better performance, although the average accuracy results are still similar to those obtained with CSP. Therefore, although these divergence-based methods are not yet a practical alternative to CSP, this line of research is in its infancy, and divergence-based methods can have an underlying potential for improvements in performance that remains to be explored.

## Figures and Tables

**Table 1:** Computational burden of the considered algorithms, which are sorted in increasing value of their respective execution times without using cross-validation. FBCSP, filter bank CSP; ITFE, information theoretic feature extraction.

Algorithm	Time (s)
CSP	0.0017
FBCSP	0.0050
ITFE	0.3070
Sub-LD	1.0538
DivCSP	4.6696
